# Serum Osteoprotegerin Is a Potential Biomarker of Insulin Resistance in Chinese Postmenopausal Women with Prediabetes and Type 2 Diabetes

**DOI:** 10.1155/2017/8724869

**Published:** 2017-01-31

**Authors:** Peng Duan, Min Yang, Meilin Wei, Jia Liu, Ping Tu

**Affiliations:** ^1^Department of Endocrinology and Metabolism, Nanchang Key Laboratory of Diabetes, The Third Hospital of Nanchang, No. 2 Xiangshan South Road, Nanchang, Jiangxi 330009, China; ^2^Department of Finance, Nanchang Normal University, No. 889 Ruixiang Road, Nanchang, Jiangxi 330009, China

## Abstract

The aim of this study is to investigate the circulating OPG levels in postmenopausal women with diabetes and prediabetes and explore the relationships between serum OPG and insulin resistance. A total of 271 unrelated Chinese postmenopausal women were recruited in this study. The subjects were divided into type 2 diabetes mellitus (T2DM) group (*n* = 93), impaired glucose regulation (IGR) (*n* = 90), and normal glucose regulation group (NGR) (*n* = 88), according to different glucose regulation categories. Serum OPG levels were measured by enzyme-linked immunosorbent assay. The serum OPG concentration in NGR group, 151.00 ± 45.72 pg/mL, was significantly lower than that in IGR group (169.28 ± 64.91 pg/mL) (*p* = 0.031) and T2DM group (183.20 ± 56.53 pg/mL) (*p* < 0.01), respectively. In multiple linear regression analysis, HOMA-IR, age, 2hPG, AST, ALP, and eGFR were found to be independent predictors of OPG. Increased serum OPG levels (OR = 1.009, *p* = 0.006) may be a risk factor for insulin resistance. The present study suggests that OPG might be implicated in the pathogenesis of diabetes and is a potential biomarker of insulin resistance in subjects with diabetes and prediabetes.

## 1. Introduction

Osteoprotegerin (OPG) is a secreted glycoprotein that belongs to the tumor necrosis factor receptor superfamily. OPG is known to act as a decoy receptor for the receptor activator of nuclear factor-*κ*B (NF-*κ*B) ligand (RANKL), which stimulates osteoclastogenesis and bone resorption by binding to its receptor activator of NF-*κ*B (RANK). OPG could prevent RANKL from binding to RANK on osteoclasts, thus, inhibiting osteoclast differentiation and bone resorption [1].

It has been well recognized that OPG could regulate bone metabolism through essential roles in the formation, activation, and survival of osteoclasts [2]. In addition, OPG plays an important role in atherosclerosis, arterial calcification, and vascular disease [3]. OPG is highly expressed in the liver, kidney, bone marrow, and other tissues and produced by a variety of cell types including endothelial cells and smooth muscle cells [4]. Clinical studies indicate that serum OPG concentrations are associated with coronary artery disease, vascular calcification, diabetic complications, and cardiovascular mortality [5–7]. Recent studies suggest that OPG may be a new marker for diabetic cardiovascular complications and atherosclerosis [8].

Elevated concentrations of OPG have been reported in diabetic individuals and were independently associated with the diabetic microvascular complications [9]. However, the serum OPG concentrations in individuals with prediabetes, such as impaired glucose tolerance, have rarely been studied. We hypothesized that serum OPG levels might be increased at the stage of prediabetes, and serum OPG may be a potential biomarker in diabetes and prediabetes. Therefore, we conducted a study to investigate the changes in serum levels of OPG in Chinese postmenopausal women with diabetes and prediabetes and explore the relationships between serum OPG and insulin resistance, body mass index, lipid profile, and blood glucose.

## 2. Subjects and Methods

### 2.1. Study Population

A total of 271 unrelated southern Han Chinese postmenopausal women were recruited from the database of our previous female osteoporosis study [10] from February 1, 2013, to November 30, 2013. Standard oral glucose tolerance testing was performed in all the subjects except those who had already been diagnosed with type 2 diabetes. The subjects were divided into type 2 diabetes (T2DM) group (*n* = 93), impaired glucose regulation (IGR) group (*n* = 90), and normal glucose regulation (NGR) group (*n* = 88), according to different glucose regulation categories.

Diabetes was defined using the World Health Organization 1999 criteria: (1) fasting plasma glucose (FPG) ≥ 7.0 mmol/L or (2) 2 h postprandial plasma glucose (2hPG) in oral glucose tolerance test ≥ 11.1 mmol/L or (3) typical symptoms with random plasma glucose ≥ 11.1 mmol/L. Impaired glucose regulation, also termed as prediabetes, was defined as follows: 6.1 mmol/L ≤ FPG < 7.0 mmol/L and/or 7.8 mmol/L ≤ 2hPG < 11.1 mmol/L. The criteria for normal glucose regulation were defined as follows: FPG < 6.1 mmol/L and 2hPG < 7.8 mmol/L.

Exclusion criteria included subjects who suffered from diseases associated with disordered glucose metabolism, such as chronic liver disease, severe kidney disease, hypothyroidism, and pituitary or adrenal diseases. Participants who had taken glucocorticosteroid or bisphosphonates within the past 3 months and had a fracture within 1 year were also excluded. In the study, 12 participants had taken calcium and/or vitamin D. In the diabetes group, the average duration of diabetes was 4.0 ± 4.5 years, 55 individuals were newly diagnosed with diabetes without any medication, 2 patients were treated with insulin, and 36 patients were treated with oral hypoglycemic agents, such as metformin, sulfonylureas, and acarbose, but no thiazolidinediones.

Demographic information was collected by an interview through a questionnaire, such as age, smoking history, alcohol intake, and a detailed medical history, especially the history of diabetes. The study was approved by the ethics committee of the Third Hospital of Nanchang, and the subjects provided written informed consent before participation in the study.

### 2.2. Anthropometric Measurements

Height and weight were measured to the nearest 1 cm and 0.1 kg, respectively. Body mass index (BMI) was calculated [formula: BMI = body weight (kg)/height^2^ (m^2^)]. Waist circumference and hip circumference (to the nearest 1 cm) were measured, and waist-to-hip ratio (WHR) was calculated [formula: WHR = waist circumference (cm)/hip circumference (cm)]. Blood pressure and pulse rates were measured in the sitting position using electronic sphygmomanometer Omron, HEM-7200 (Omron Inc., Tokyo, Japan). Body fat percentage was detected using electronic body fat measuring instrument Omron, HBF-375 (Omron Inc., Tokyo, Japan). Bone mineral densities at the lumbar spine (L_2_–L_4_) and femoral neck were measured by dual X-ray absorptiometry (DXA, GE-Lunar Prodigy, Waukesha, WI, USA).

### 2.3. Biochemical Assays

Blood samples were taken after an overnight fast. Measurements of serum biochemical parameters, such as FPG, 2hPG, alanine aminotransferase (ALT), aspartate aminotransferase (AST), alkaline phosphatase (ALP), blood urea nitrogen (BUN), creatinine (Cr), high-density lipoprotein cholesterol (HDL-C), low-density lipoprotein cholesterol (LDL-C), total cholesterol (TC), triglycerides (TG), and blood uric acid (URCA), were performed using commercially available kits (Siemens Inc., Erlangen, Germany) on the day of the collection. Subsequently, serum was separated, stored at −80°C, until OPG analysis. Serum insulin was measured using an electrochemiluminescence immunoassay (Roche Diagnostics, Indianapolis, USA), while glycated hemoglobin (HbA1c) was detected by high-performance liquid chromatography (Bio-Rad Inc., CA, USA). Serum OPG levels were measured by enzyme-linked immunosorbent assay kit (RayBiotech, Norcross, GA, USA). All of the samples were assayed blindly, in duplicate, and in random order. The sensitivity of the kit was minimally 1 pg/mL and the mean intra- and interassay coefficients of variation were below 10%.

Insulin resistance was determined by homeostasis model assessment (HOMA-IR), and HOMA-IR index was calculated [formula: fasting plasma glucose (mmol/L) × fasting serum insulin (mU/mL)/22.5]. According to previous studies, HOMA-IR index > 1.8 has been suggested as a cut-off point to indicate increased insulin resistance [11]. The homeostasis model assessment of *β*-cell function (HOMA-B) (%) was calculated using the formula: HOMA-B = (20 × fasting insulin)/(fasting glucose − 3.5) [12]. The quantitative insulin-sensitivity check index (QUICKI) was estimated according to the formula: QUICKI = 1/[log(fasting insulin in mU/mL) + log(fasting glucose in mg/dl)] [13]. The abbreviated MDRD equation was used to estimate glomerular filtration rate (eGFR) as 186 × [plasma creatinine^− 1.154^] × (age)^− 0.203^ × (0.742 if female) [14].

### 2.4. Statistical Analysis

Data are reported as means ± standard deviation or median (25th–75th percentile). SPSS 19.0 (SPSS Inc., Chicago, IL, USA) was used to perform the statistical analysis. Normality was tested for via the Kolmogorov-Smirnov test and if violated, a natural logarithmic transformation (e.g., HOMA-IR) was used where possible. Statistical comparisons between groups were made using one-way ANOVA with post hoc comparisons for normally distributed variables [15]. For non-normally distributed variables (e.g., HbA1c, HOMA-IR), nonparametric comparisons were made using Kruskal-Wallis test. The statistical differences in means of average serum OPG levels between each group were determined by analysis of covariance, which was adjusted for the confounding factors in different models. The bivariate correlations between OPG and other parameters were determined by Spearman's correlation analysis. Stepwise multilinear regression analysis was performed in order to study the independent variables that may affect OPG values. Logistic regression was used to assess the association between insulin resistance and serum parameters (e.g., OPG, TC, and TG) and other potential independent variables (e.g., age, BMI, and HbA1c). Statistical significance is defined as a *p* value < 0.05 on two-tailed testing.

## 3. Results

### 3.1. Characteristics of Participants

The basic characteristics of T2DM group (*n* = 93), IGR group (*n* = 90), and NGR group (*n* = 88) were shown in Table 1. There was no significant difference in age, DBP, pulse rates, height, hip circumference, body fat percentage, AST, BNU, Cr, eGFR, and LDL-C between these groups (all *p* > 0.05). The serum OPG concentration in NGR group, 151.00 ± 45.72 pg/mL, was significantly lower than that in IGR group (169.28 ± 64.91 pg/mL) (*p* = 0.031) and T2DM group (183.20 ± 56.53 pg/mL) (*p* < 0.001), respectively. The serum OPG level in IGR group was lower than T2DM group, although there was no statistically significant difference (*p* = 0.096). The serum OPG levels in IGR group and T2DM group are still higher than that in NGR group even after adjustment for other confounding factors in different models (Table 2). In addition, the weight, waist-to-hip ratio, ALT, URCA, TG, FPG, 2hPG, FINS, HbA1c, HOMA-IR, and lumbar and femoral neck BMD in IGR and T2DM groups were significantly higher than those in NGR group (all *p* < 0.05).

### 3.2. Relationship between Serum OPG Levels and Clinical Parameters

Bivariate correlation analysis of serum OPG levels with clinical parameters was performed (Table 3). Serum OPG levels showed a significantly positive correlation with HOMA-IR (*r* = 0.134, *p* = 0.027), waist-to-hip ratio, body fat percentage, ALP, 2hPG, and HbA1c (all *p* < 0.05) but significantly negative correlation with lumbar and femoral neck BMD (*p* = 0.002 and *p* = 0.032).

In stepwise multiple linear regression analysis with OPG as a dependent variable, age, body fat percentage, waist-to-hip ratio, BMI, SBP, DBP, ALT, AST, ALP, eGFR, TC, TG, HDL-C, LDL-C, 2hPG, HbA1c, and HOMA-IR were added to the model. Finally, HOMA-IR, age, 2hPG, AST, ALP, and eGFR were found to be independent predictors of serum OPG (all *p* < 0.05) (Table 4).

In logistic regression analyses with insulin resistance as the dependent variable, OPG, BMI, ALT, URCA, HDL-C, LDL-C, and HbA1c were significantly associated with insulin resistance (all *p* < 0.05). As shown in Table 5, increased serum OPG levels (OR = 1.009, CI 95% = 1.003–1.015, *p* = 0.006) may be a risk factor for insulin resistance in postmenopausal women.

## 4. Discussion

In the present study, we demonstrated that circulating OPG concentrations were increased in Chinese postmenopausal women with diabetes and prediabetes. Moreover, serum OPG levels showed significant correlation with insulin resistance.

HOMA-IR was used as a surrogate measure of insulin resistance in our study. Although HOMA-IR was not the gold standard for assessment of insulin sensitivity, it was a clinically useful index used in many studies [16]. Population-based studies for defining cut-off values of HOMA-IR for the diagnosis of insulin resistance have been conducted in different geographic areas. At present, there is no national survey data about the cut-off values of HOMA-IR in Chinese population. Thus, we defined HOMA-IR index >1.8 as a cut-off point to indicate increased insulin resistance according to previous studies [11, 17, 18]. Studies have indicated that there are age and gender-specific differences in HOMA-IR levels, with increased levels in women over fifty years of age. Although the cut-off point 1.8 may not be suitable for the Chinese population, it can provide a useful reference point for our study when there is no available data for the Chinese population.

Previous reports had reported similar results about the association between serum OPG levels and HOMA-IR in subjects with type 2 diabetes mellitus [19] and Caucasian obese population [20]. In contrast, several studies have shown a negative association between OPG and HOMA-IR in obese African women [21] and Turkish population [22]. Besides, two studies in healthy Korean women and Irish population did not find any relationship between these two parameters [23, 24]. This result has long been a matter of controversy in the literature, possibly because of different methodologies and sample sizes of the study populations. Our findings are in line with a recent report by Niu et al. [25]; the authors found serum OPG levels were significantly associated with HOMA-IR in Chinese population, and serum OPG levels were significantly higher in subjects with impaired glucose regulation and diabetes than in those with normal glucose regulation.

OPG could be produced by a variety of cells of the cardiovascular system, including vascular smooth muscle cells and endothelial cells, and OPG represents a protective factor for vascular system [26]. For example, OPG has been reported to increase cell proliferation of human artery and vein endothelial cells, maybe via inducing phosphorylation of extracellular signal-regulated kinases1/2 and protein kinase B in those cells, which are similar to the effects of those growth factors such as fibroblast growth factor and vascular endothelial growth factor in the endothelial cells [27–30]. It is suggested that increased serum OPG levels in subjects with diabetes have been interpreted as an insufficient compensatory self-defensive response to prevent vascular endothelial dysfunction and the progression of atherosclerosis [31]. Thus, some studies suggest that increased OPG production represents an early event in the natural history of diabetes, possibly contributing to diabetes-associated vascular endothelial cell dysfunction [32].

In our study, serum OPG levels showed significant association with insulin resistance, but the mechanisms underlying the association are currently unclear. It is thought that inflammation may link OPG to insulin resistance. Insulin resistance is a hallmark of type 2 diabetes mellitus and regarded as a chronic low-grade systemic inflammation [33]. It has been demonstrated that OPG was positively correlated with inflammatory markers and played a causal role in the pathogenesis of inflammation [34]. OPG/RANK/RANKL system is believed to be associated with the regulation of inflammatory and immune responses and directly contributed to the regulation of proinflammatory cytokine production in macrophages [35]. Additionally, it is well established that the OPG/RANK/RANKL system could activate the NF-*κ*B pathway and its downstream players [36], which are closely related to the pathogenesis of insulin resistance [37]. Thus, OPG may have a role in insulin resistance through NF-*κ*B pathway. Recently, evidence has indicated that the OPG/RANK/RANKL system may have a potential role in the pathogenesis of diabetes; blocking this pathway improved hepatic insulin resistance and prevented the development of diabetes mellitus [38].

It has been recognized that the OPG is a cytokine that increases the mineral density and volume of bone tissue by decreasing the number of active osteoclasts. In our study, bivariate analysis showed an inverse correlation between OPG levels and BMD in postmenopausal women. The result was teleologically interpreted as a counter-regulatory mechanism in order to prevent further bone loss. A significantly negative correlation between circulating levels of OPG and BMD was also reported in two studies in patients with type 1 and 2 diabetes mellitus [39, 40]. However, a positive correlation between serum levels of OPG and BMD was found in postmenopausal women by Nabipour et al. [41]. In addition, several other studies have not demonstrated an association between serum OPG concentration and BMD [42]. The reason for these conflicting results is unclear but may lie in differences in populations or assays studied or in incomplete control for confounding variables.

Additionally, we found serum OPG levels were positively correlated with age, 2hPG, HbA1c, AST, ALP, and eGFR through linear regression analysis. These findings were consistent with evidences from previous studies that found serum OPG levels were associated with several clinical and biochemical parameters [43]. The increasing serum concentrations of OPG with aging could be explained by a compensatory mechanism to counteract the acceleration of bone resorption [44]. Serum OPG levels were significantly associated with AST, ALP, and eGFR, which were the markers of liver and renal function, and these results may be interpreted as follows: OPG was highly expressed in the liver and kidney, and there were significant associations between high OPG serum levels and liver and kidney dysfunctions [45, 46]. The serum OPG was significantly increased in patients with chronic kidney disease. Recent evidences suggest that inflammation, secondary hyperparathyroidism, disorder of bone metabolism, vascular calcifications, and atherosclerosis may play important roles in this process [47].

The main limitation of our study is small sample size; our current findings need to be confirmed in further large population studies. In addition, serum OPG concentrations are gender-specific; for example, women have higher circulating OPG levels than men. We conducted a study in a select group of Chinese postmenopausal women, and the subjects were recruited from the database of our previous female osteoporosis study. Thus, future researches in randomly selected and different gender population are needed. Furthermore, the exact mechanisms underlying the observed associations in this study remain to be determined.

## 5. Conclusions

The current study demonstrates that serum OPG level is elevated in postmenopausal women with diabetes and prediabetes and significantly associated with insulin resistance. These findings suggest that OPG might be implicated in the pathogenesis of diabetes and is a potential biomarker of insulin resistance in subjects with diabetes and prediabetes.

## Figures and Tables

**Table 1:** Clinical and biochemical characteristics of the study subjects.

Variable	NGR group (*n* = 88)	IGR group (*n* = 90)	T2DM group (*n* = 93)
Age (year)	62.83 ± 8.57	62.38 ± 9.02	63.81 ± 8.69
SBP (mmHg)	138.40 ± 20.76	141.80 ± 19.54	147.62 ± 21.06^a^
DBP (mmHg)	80.06 ± 13.61	80.9 ± 12.43	80.97 ± 12.93
Pulse rates (beats/min)	87 ± 13	88 ± 13	87 ± 11
Height (cm)	153.10 ± 5.50	154.03 ± 6.28	153.71 ± 6.23
Weight (kg)	57.24 ± 9.25	60.36 ± 10.41^a^	60.80 ± 8.59^a^
BMI (kg/m^2^)	24.40 ± 3.56	25.38 ± 3.67	25.70 ± 3.06^a^
Waist circumference (cm)	77.33 ± 9.16	79.84 ± 9.38	81.95 ± 8.86^a^
Hip circumference (cm)	91.98 ± 7.78	92.53 ± 8.99	93.51 ± 7.84
Waist-to-hip ratio	0.84 ± 0.06	0.86 ± 0.06^a^	0.88 ± 0.06^a^
Body fat percentage (%)	37.34 ± 3.66	37.69 ± 4.11	37.81 ± 3.84
ALT (U/L)	22.0 (17.0–22.8)	28.0 (21.0–41.3)^a^	27.0 (17.5–42.0)^a^
AST (U/L)	28.0 (24.0–35.5)	30.0 (23.8–34.0)	26.0 (21.0–34.5)
ALP (U/L)	77.0 (64.8–92.8)	82.0 (65.8–96.5)	82.0 (70.0–96.0)
BUN (mmol/L)	4.76 (4.03–5.81)	4.67 (3.82–5.41)	5.03 (4.02–5.98)
Cr (*μ*mol/L)	62.0 (69.0–77.0)	70.0 (61.8–77.0)	67.0 (60.0–78.0)
URCA (*μ*mol/L)	282.0 (243.8–334.0)	316.5 (283.8–376.5)^a^	312.0 (263.5–363.5)^a^
TC (mmol/L)	5.38 ± 1.13	5.62 ± 1.14	5.81 ± 1.52^a^
TG (mmol/L)	1.07 (0.85–1.34)	1.34 (1.08–1.85)^a^	1.40 (1.05–1.93)^a^
HDL-C (mmol/L)	1.58 (1.38–1.91)	1.47 (1.32–1.73)	1.41 (1.26–1.63)^a^
LDL-C (mmol/L)	2.94 (2.64–3.48)	3.24 (2.81–3.75)	3.22 (2.71–3.70)
FPG (mmol/L)	4.60 (4.18–4.90)	5.19 (4.99–5.89)^a^	6.16 (6.89–8.98)^a, b^
2hPG (mmol/L)	5.62 (4.82–6.22)	8.52 (7.98–9.53)^a^	13.46 (11.61–16.77)^a, b^
HbA1c (%)	5.5 (5.4–5.7)	6.0 (5.6–6.3)^a^	7.4 (6.1–8.0)^a, b^
FINS (mIU/L)	8.00 (4.94–11.18)	11.55 (7.25–15.48)^a^	10.99 (7.39–15.34)^a^
HOMA-IR index	1.54 (1.02–2.22)	2.39 (1.80–3.78)^a^	3.48 (2.33–5.06)^a, b^
HOMA-B	155.60 (86.03–253.09)	126.30 (76.02–222.09)	64.27 (40.22–107.40)^a, b^
QUICKI	0.362 ± 0.032	0.334 ± 0.034^a^	0.319 ± 0.027^a, b^
Lumbar BMD (mg/cm^2^)	0.866 ± 0.173	0.903 ± 0.168	0.937 ± 0.146^a^
Femoral neck BMD (mg/cm^2^)	1.006 ± 0.174	1.053 ± 0.186	1.074 ± 0.155^a^
Serum OPG (pg/mL)	151.00 ± 45.72	169.28 ± 64.91^a^	183.20 ± 56.53^a^
eGFR	81.98 ± 17.61	80.96 ± 18.84	81.72 ± 18.47
Smoking history (%)	1 (1.14)	2 (2.22)	2 (2.15)
Alcohol intake (%)	3 (3.41)	3 (3.33)	2 (2.15)
Calcium and vitamin D supplements (%)	3 (3.41)	5 (5.56)	4 (4.30)

Data are presented as means ± SD or median (25th–75th percentile). ^a^*p* < 0.05 versus NGT group; ^b^*p* < 0.05 versus IGR group.

2hPG, 2 h postprandial plasma glucose; ALP, alkaline phosphatase; ALT, alanine aminotransferase; AST, aspartate aminotransferase; BMD, bone mineral density; BMI, body mass index; BUN, blood urea nitrogen; Cr, creatinine; eGFR, estimate glomerular filtration rate; FINS, fasting insulin; FPG, fasting plasma glucose; HbA1c, glycated hemoglobin; HDL-C, high-density lipoprotein cholesterol; HOMA-B, homeostasis model assessment of *β*-cell function; HOMA-IR, homeostasis model of assessment for insulin resistance; IGR, impaired glucose regulation; LDL-C, low-density lipoproteincholesterol; NGR, normal glucose regulation; OPG, osteoprotegerin; QUICKI, quantitative insulin sensitivity check index; SBP, systolic blood pressure; DBP, diastolic blood pressure; T2DM, type 2 diabetes; TC, total cholesterol; TG, triglycerides; URCA, blood uric acid.

**Table 2:** Comparsion of serum osteoprotegerin levels with different glucose regulation.

Model	Serum osteoprotegerin (pg/mL)			*p* value^d^	*p* value^e^	*p* value^f^
	NGR group (*n* = 88)	IGR group (*n* = 90)	T2DM group (*n* = 93)			
Model 1^a^	151.00 ± 4.87	169.28 ± 6.84	183.20 ± 5.86	0.031	<0.001	0.096
Model 2^b^	153.69 ± 5.33	170.75 ± 5.19	179.23 ± 5.16	0.023	0.001	0.247
Model 3^c^	154.54 ± 5.24	170.61 ± 5.05	178.56 ± 4.98	0.031	0.001	0.261

Serum OPG data is presented as means ± SE. IGR, impaired glucose regulation; NGR, normal glucose regulation; T2DM, type 2 diabetes.

^a^Unadjusted; ^b^adjusted for age, body mass index, waist-to-hip ratio, and body fat percentage; ^c^adjusted for age, body mass index, waist-to-hip ratio, body fat percentage, eGFR, blood uric acid, aspartate aminotransferase, and alkaline phosphatase; ^d^NGT group versus IGR group; ^e^NGT group versus T2DM group; ^f^IGR group versus T2DM group.

**Table 3:** Bivariate correlation analysis between study parameters and serum osteoprotegerin levels.

Parameters	Bivariate correlation	
	*r*	*p* value
Age	0.453	<0.001
Height	−0.115	0.058
Weight	−0.082	0.178
BMI	−0.036	0.552
Waist circumference	0.078	0.203
Hip circumferences	−0.005	0.793
Waist-to-hip ratio	0.125	0.039
Body fat percentage	0.213	<0.001
ALT	−0.014	0.823
AST	0.086	0.157
ALP	0.175	0.004
BUN	0.025	0.688
Cr	0.193	0.001
Calcium	−0.001	0.981
URCA	0.046	0.451
TC	−0.006	0.924
TG	0.027	0.660
HDL-C	0.012	0.847
LDL-C	−0.077	0.207
FPG	0.093	0.133
2hPG	0.270	<0.001
HbA1c	0.214	<0.001
FINS	0.106	0.080
Ln HOMA-IR	0.134	0.027
HOMA-B	−0.037	0.546
QUICKI	−0.194	0.001
eGFR	−0.331	<0.001
Lumbar BMD	−0.189	0.002
Femoral neck BMD	−0.130	0.032

2hPG, 2 h postprandial plasma glucose; ALP, alkaline phosphatase; ALT, alanine aminotransferase; AST, aspartate aminotransferase; BMD, bone mineral density; BMI, body mass index; BUN, blood urea nitrogen; Cr, creatinine; eGFR, estimate glomerular filtration rate; FINS, fasting insulin; FPG, fasting plasma glucose; HbA1c, glycated hemoglobin; HDL-C, high-density lipoprotein cholesterol; HOMA-B, homeostasis model assessment of *β*-cell function; LDL-C, low-density lipoproteincholesterol; Ln HOMA-IR, natural logarithmic transformation homeostasis model of assessment for insulin resistance index; OPG, osteoprotegerin; QUICKI, quantitative insulin sensitivity check index; SBP, systolic blood pressure; SDP, diastolic blood pressure; TC, total cholesterol; TG, triglycerides; URCA, blood uric acid.

**Table 4:** Multiple linear regression analysis with osteoprotegerin as a dependent variable.

Parameters	*β*	*p* value
Age	2.747	<0.001
HOMA-IR	2.591	0.024
eGFR	−0.457	0.010
AST	0.575	0.001
ALP	0.362	0.003
2hPG	2.289	0.001

2hPG, 2 h postprandial plasma glucose; ALP, alkaline phosphatase; AST, aspartate aminotransferase; eGFR, estimate glomerular filtration rate; HOMA-IR, homeostasis model of assessment for insulin resistance.

**Table 5:** Stepwise logistic regression analysis with insulin resistance as a dependent variable.

Parameters	Odds ratio	95% confidence interval	*p* value
OPG	1.009	1.003–1.015	0.006
BMI	1.238	1.112–1.378	<0.001
URCA	1.005	1.001–1.010	0.020
HDL-C	0.351	0.132–0.930	0.035
LDL-C	1.724	1.162–2.558	0.007
HbA1c	2.048	1.368–3.065	0.001
ALT	1.024	1.005–1.043	0.012

ALT, alanine aminotransferase; BMI, body mass index; HbA1c, glycated hemoglobin; HDL-C, high-density lipoprotein cholesterol; LDL-C, low-density lipoproteincholesterol; OPG, osteoprotegerin; URCA, blood uric acid.

## References

[1] Martin T. J., Sims N. A. (2015). RANKL/OPG; critical role in bone physiology. *Reviews in Endocrine & Metabolic Disorders*.

[2] Liu W., Zhang X. (2015). Receptor activator of nuclear factor-*κ*B ligand (RANKL)/RANK/osteoprotegerin system in bone and other tissues. *Molecular Medicine Reports*.

[3] Augoulea A., Vrachnis N., Lambrinoudaki I. (2013). Osteoprotegerin as a marker of atherosclerosis in diabetic patients. *International Journal of Endocrinology*.

[4] Esteghamati A., Aflatoonian M., Rad M. V. (2015). Association of osteoprotegerin with peripheral artery disease in patients with type 2 diabetes. *Archives of Cardiovascular Diseases*.

[5] Pérez de Ciriza C., Lawrie A., Varo N. (2015). Osteoprotegerin in cardiometabolic disorders. *International Journal of Endocrinology*.

[6] Lewis J. R., Lim W. H., Ueland T. (2015). Elevated circulating osteoprotegerin and renal dysfunction predict 15-year cardiovascular and all-cause mortality: a prospective study of elderly Women. *PLoS ONE*.

[7] Morisawa T., Nakagomi A., Kohashi K. (2015). Osteoprotegerin is associated with endothelial function and predicts early carotid atherosclerosis in patients with coronary artery disease. *International Heart Journal*.

[8] Lambrinoudaki I., Tsouvalas E., Vakaki M. (2013). Osteoprotegerin, soluble receptor activator of nuclear factor-*κ*B ligand, and subclinical atherosclerosis in children and adolescents with type 1 diabetes mellitus. *International Journal of Endocrinology*.

[9] Blázquez-Medela A. M., López-Novoa J. M., Martínez-Salgado C. (2011). Osteoprotegerin and diabetes-associated pathologies. *Current Molecular Medicine*.

[10] Tu P., Duan P., Zhang R. S. (2015). Polymorphisms in genes in the RANKL/RANK/OPG pathway are associated with bone mineral density at different skeletal sites in post-menopausal women. *Osteoporosis International*.

[11] Maser R. E., Lenhard M. J., Sneider M. B., Pohlig R. T. (2015). Osteoprotegerin is a better serum biomarker of coronary artery calcification than osteocalcin in type 2 diabetes. *Endocrine Practice*.

[12] Matthews D. R., Hosker J. P., Rudenski A. S., Naylor B. A., Treacher D. F., Turner R. C. (1985). Homeostasis model assessment: insulin resistance and *β*-cell function from fasting plasma glucose and insulin concentrations in man. *Diabetologia*.

[13] Katz A., Nambi S. S., Mather K. (2000). Quantitative insulin sensitivity check index: a simple, accurate method for assessing insulin sensitivity in humans. *The Journal of Clinical Endocrinology & Metabolism*.

[14] Levey A. S., Bosch J. P., Lewis J. B., Greene T., Rogers N., Roth D. (1999). A more accurate method to estimate glomerular filtration rate from serum creatinine: a new prediction equation. Modification of Diet in Renal Disease Study Group. *Annals of Internal Medicine*.

[15] Li Y., Wang K., Zou Q. Y., Magness R. R., Zheng J. (2015). 2,3,7,8-Tetrachlorodibenzo-p-dioxin differentially suppresses angiogenic responses in human placental vein and artery endothelial cells. *Toxicology*.

[16] Cicero A. F., Rosticci M., Parini A. (2015). Short-term effects of a combined nutraceutical of insulin-sensitivity, lipid level and indexes of liver steatosis: a double-blind, randomized, cross-over clinical trial. *Nutrition Journal*.

[17] Esteghamati A., Ashraf H., Esteghamati A. R. (2009). Optimal threshold of homeostasis model assessment for insulin resistance in an Iranian population: the implication of metabolic syndrome to detect insulin resistance. *Diabetes Research and Clinical Practice*.

[18] Gayoso-Diz P., Otero-González A., Rodriguez-Alvarez M. X., García F., De Francisco A., Quintela A. G. (2013). Insulin resistance (HOMA-IR) cut-off values and the metabolic syndrome in a general adult population: effect of gender and age: EPIRCE cross-sectional study. *BMC Endocrine Disorders*.

[19] Yaturu S., Rains J., Jain S. K. (2008). Relationship of elevated osteoprotegerin with insulin resistance, CRP, and TNF-alpha levels in men with type 2 diabetes. *Cytokine*.

[20] Gannagé-Yared M. H., Yaghi C., Habre B. (2008). Osteoprotegerin in relation to body weight, lipid parameters, insulin sensitivity, adipocytokines, and C-reactive protein in obese and non-obese young individuals: results from both cross-sectional and interventional study. *European Journal of Endocrinology*.

[21] Ayina Ayina C. N., Sobngwi E., Essouma M. (2015). Osteoprotegerin in relation to insulin resistance and blood lipids in sub-Saharan African women with and without abdominal obesity. *Diabetology & Metabolic Syndrome*.

[22] Ugur-Altun B., Altun A., Gerenli M., Tugrul A. (2005). The relationship between insulin resistance assessed by HOMA-IR and serum osteoprotegerin levels in obesity. *Diabetes Research and Clinical Practice*.

[23] Oh E. S., Rhee E. J., Oh K. W. (2005). Circulating osteoprotegerin levels are associated with age, waist-to-hip ratio, serum total cholesterol, and low-density lipoprotein cholesterol levels in healthy Korean women. *Metabolism*.

[24] O’Sullivan E. P., Ashley D. T., Davenport C. (2013). A comparison of osteoprotegerin with adiponectin and high-sensitivity C-reactive protein (hsCRP) as a marker for insulin resistance. *Metabolism*.

[25] Niu Y., Yang Z., Li X., Zhang W., Lu S., Zhang H., Chen X. (2015). Association of osteoprotegerin with impaired glucose regulation and microalbuminuria: the REACTION study. *BMC Endocrine Disorders*.

[26] Wang H. H., Xiang G. D. (2015). Changes of plasma concentration of osteoprotegerin and its association with endothelial dysfunction before and after hypouricemic therapy in patients with hyperuricemia. *Modern Rheumatology*.

[27] Jiang Y. Z., Wang K., Li Y. (2013). Enhanced cellular responses and distinct gene profiles in human fetoplacental artery endothelial cells under chronic low oxygen. *Biology of Reproduction*.

[28] Jiang Y. Z., Wang K., Li Y. (2013). Transcriptional and functional adaptations of human endothelial cells to physiological chronic low oxygen. *Biology of Reproduction*.

[29] Jiang Y. Z., Li Y., Wang K. (2014). Distinct roles of HIF1A in endothelial adaptations to physiological and ambient oxygen. *Molecular and Cellular Endocrinology*.

[30] McGonigle J. S., Giachelli C. M., Scatena M. (2009). Osteoprotegerin and RANKL differentially regulate angiogenesis and endothelial cell function. *Angiogenesis*.

[31] Wang S. T., Zhang C. Y., Zhang C. M., Zhang C. M., Rong W. (2015). The plasma osteoprotegerin level and osteoprotegerin expression in renal biopsy tissue are increased in type 2 diabetes with nephropathy. *Experimental and Clinical Endocrinology & Diabetes*.

[32] Secchiero P., Corallini F., Pandolfi A. (2006). An increased osteoprotegerin serum release characterizes the early onset of diabetes mellitus and may contribute to endothelial cell dysfunction. *The American Journal of Pathology*.

[33] Chen L., Chen R., Wang H., Liang F. (2015). Mechanisms linking inflammation to insulin resistance. *International Journal of Endocrinology*.

[34] Bernardi S., Fabris B., Thomas M. (2014). Osteoprotegerin increases in metabolic syndrome and promotes adipose tissue proinflammatory changes. *Molecular and Cellular Endocrinology*.

[35] Shimamura M., Nakagami H., Osako M. K. (2014). OPG/RANKL/RANK axis is a critical inflammatory signaling system in ischemic brain in mice. *Proceedings of the National Academy of Sciences of the United States of America*.

[36] Kondegowda N. G., Fenutria R., Pollack I. R. (2015). Osteoprotegerin and denosumab stimulate human beta cell proliferation through inhibition of the receptor activator of NF-*κ*B ligand pathway. *Cell Metabolism*.

[37] Weidemann A., Lovas A., Rauch A. (2016). Classical and alternative NF-*κ*B signaling cooperate in regulating adipocyte differentiation and function. *International Journal of Obesity*.

[38] Kiechl S., Wittmann J., Giaccari A. (2013). Blockade of receptor activator of nuclear factor-*κ*B (RANKL) signaling improves hepatic insulin resistance and prevents development of diabetes mellitus. *Nature Medicine*.

[39] Loureiro M. B., Ururahy M. A., Freire-Neto F. P. (2014). Low bone mineral density is associated to poor glycemic control and increased OPG expression in children and adolescents with type 1 diabetes. *Diabetes Research and Clinical Practice*.

[40] Suzuki K., Kurose T., Takizawa M. (2005). Osteoclastic function is accelerated in male patients with type 2 diabetes mellitus: the preventive role of osteoclastogenesis inhibitory factor/osteoprotegerin (OCIF/OPG) on the decrease of bone mineral density. *Diabetes Research and Clinical Practice*.

[41] Nabipour I., Larijani B., Vahdat K. (2009). Relationships among serum receptor of nuclear factor-kappaB ligand, osteoprotegerin, high-sensitivity C-reactive protein, and bone mineral density in postmenopausal women: osteoimmunity versus osteoinflammatory. *Menopause*.

[42] Kudlacek S., Schneider B., Woloszczuk W., Pietschmann P., Willvonseder R., Austrian Study Group on Normative Values of Bone Metabolism (2003). Serum levels of osteoprotegerin increase with age in a healthy adult population. *Bone*.

[43] Altinova A. E., Toruner F., Akturk M. (2011). Relationship between serum osteoprotegerin, glycemic control, renal function and markers of atherosclerosis in type 2 diabetes. *Scandinavian Journal of Clinical and Laboratory Investigation*.

[44] Khosla S., Arrighi H. M., Melton L. J. (2002). Correlates of osteoprotegerin levels in women and men. *Osteoporosis International*.

[45] Moschen A. R., Kaser A., Stadlmann S. (2005). The RANKL/OPG system and bone mineral density in patients with chronic liver disease. *Journal of Hepatology*.

[46] Shaarawy M., Fathy S. A., Mehany N. L., Hindy O. W. (2007). Circulating levels of osteoprotegerin and receptor activator of NF-kappaB ligand in patients with chronic renal failure. *Clinical Chemistry and Laboratory Medicine*.

[47] Spartalis M., Papagianni A. (2016). Receptor activator of nuclear factor *κ*B ligand/osteoprotegerin axis and vascular calcifications in patients with chronic kidney disease. *World Journal of Nephrology*.

